# Effects of elicitors on plant host selection by adult *Leptinotarsa decemlineata* (Coleoptera: Chrysomelidae)

**DOI:** 10.1093/jisesa/ieae112

**Published:** 2024-12-03

**Authors:** Alexander Butcher, Silvia I Rondon, Jana Lee, Ryan Paul

**Affiliations:** Oregon Integrated Pest Management Center, Crop and Soil Sciences Department, Oregon State University, Corvallis, OR, USA; Department of Horticulture, Oregon State University, Corvallis, OR, USA; Oregon Integrated Pest Management Center, Crop and Soil Sciences Department, Oregon State University, Corvallis, OR, USA; USDA-ARS Horticultural Crops Disease and Pest Management Research Unit, Corvallis, OR, USA; Department of Horticulture, Oregon State University, Corvallis, OR, USA; USDA-ARS Horticultural Crops Disease and Pest Management Research Unit, Corvallis, OR, USA

**Keywords:** behavior, volatiles, Colorado Potato Beetle, olfactometry, potato

## Abstract

The Colorado Potato Beetle, *Leptinotarsa decemlineata* Say, is the principal defoliator of potato crops globally. It is well known for its propensity to rapidly develop resistance. Thus, new control options which are resilient to the pest’s resistance capabilities are a critical need. The use of chemical ecology in integrated pest management (IPM) programs has been proposed as a means to delay resistance. Elicitors are chemical growth regulators that activate plant defenses. These plant defenses provide numerous opportunities to integrate chemical ecology into IPM programs, including changes to a plants volatile profile. In this laboratory study, we provide evidence that elicitors which mimic jasmonic acid (JA) and salicylic acid (SA) can be used to attract or repel *L. decemlineata* respectively. Adult beetles are highly attracted to potato plants sprayed with the JA mimicking elicitor Blush 2X, while plants sprayed with SA mimicking elicitor, Actigard 50WG, appear to be repellent. Additionally, residency time on plants sprayed with Actigard 50WG was significantly shorter than with control plants. The potential use of elicitors within IPM program is discussed.

## Introduction

The Colorado potato beetle *Leptinotarsa decemlineata* Say (Coleoptera: Chrysomelidae), is one of the most important insect defoliators of potatoes, *Solanum tuberosum* L. (Rondon et al. 2021). Currently, the main control tactics for this pest rely on cultural or chemical methods. The latter tactic is hampered by the development of resistance (Alyokhin et al. 2008, Crossley et al. 2019, Yang et al. 2022). Volatiles have been suggested as a novel control strategy that could help diversify the available tools in producers’ toolboxes and slow the development of resistance (Dickens 2000a, Sablon et al. 2013).

Volatiles derived from the pest species (pheromones) and plant derived volatiles (kairomones) have been used in a variety of pest management programs. They have been used for monitoring lures, mating disruption, recruitment of biocontrol agents, and as attractants in trap-and-kill systems (Martel et al. 2005, Lee 2010, Ferracini et al. 2020). Since these volatiles often play a crucial role in the insect locating hosts and mates, developing a “resistance” to them would be disadvantageous to offspring. Thus, the risk of a pest developing resistance to volatile based control tools is thought to be lower than with conventional insecticides (Witzgall et al. 2010). An aggregation pheromone, (S)-3,7-dimethyl-2-oxo-oct-6-ene-1,3-diol, produced by males of *L. decemlineata* was shown to experimentally increase pitfall trap captures by as much as 5x (Kuhar et al. 2012). However, the attraction to the aggregation pheromone is short lived and requires precise timing (Göldel et al. 2020). A female sex pheromone has been suggested by experimentation but has not yet been identified (Edwards and Seabrook 1997, Sablon et al. 2013) A blend of green leaf volatiles (GLVs) produced by potatoes have been shown to be attractive to *L. decemlineata* using electroantennography and y-tube olfactometry (Visser 1979, Dickens 2000b). However, beetles were shown to be unresponsive to individual components of this blend (Visser and Ave 1978). A synthetically produced blend of the GLVs has been shown experimentally to increase the efficacy of trap cropping (Martel et al. 2005). However, other field trails which intercropped potato with other plant species failed to show increased attraction (Thiery and Visser 1986, 1987). Plants produce different arrays of volatiles in response to damage, these are referred to as herbivore induced plant volatiles (HIPVs). These volatile profiles vary widely depending on the type of damage. Potato plants wounded by conspecific larval feeding and mechanical damage have both been shown to be more attractive than the GLVs of unwounded plants to *L. decemlineata* adults (Schutz et al. 1997). Elicitors are sprayable signal chemicals which interact with a plant’s proteome, metabolome, and transcriptome, often inducing an immune response (Bektas and Euglem 2015). Applications of methyl jasmonate, a type of elicitor, have been shown to induce changes in the volatile profile of potatoes, which attracted adult female *L. decemlineata* who were otherwise unresponsive to GLVs (Landolt et al.1999). With the potential to be inducible, long lasting, and highly attractive to adults, elicitors appear to be a promising method to utilize plant volatiles in *L. decemlineata* IPM programs.

Many synthetically produced elicitors are available and can be applied in much the same way as a conventional insecticide. Most commercially available elicitors act as structural and/or functional analogs of jasmonic acid (JA) and salicylic acid (SA) or their derivatives, such as methyl jasmonate (Bektas and Euglem 2015). However, due to the complexity of the interaction between a plant’s metabolism, immunity, and the transcriptional activity of genes, the effects of different elicitor vary widely.

The effects of other JA-type elicitors, such as prohydrojasmon, as well as SA-type elicitors, on *L. decemlineata* host selection have not been widely explored. Thus, our current study evaluated adult *L. decemlineata* attractiveness to both a prohydrojasmon-based elicitor, Blush 2X, and an SA-type elicitor, Actigard 50WG, applied to Russet Burbank potatoes.

## Materials and Methods

### Plant Material

Potato plants (cv. “Russet Burbank”) were grown in 3.8-L plastic pots at the USDA-ARS greenhouse in Corvallis, OR. Plants were grown under ambient light conditions at approximately 27 ± 2 °C, 14 h of light, and 75% ± 15% R.H. Tubers were obtained from the Oregon State University Potato Breeding Program. Tubers were removed from cold storage in early spring and allowed to sprout in paper bags. Sprouted tubers were cut into approximately 0.06 kg pieces. Each piece was planted at a 15 cm depth in a pot containing a potting mix consisting of 30% sand, 20% perlite, and 50% sterilized compost. Irrigation was initiated after plants emerged; plants were watered every three days. Plants at the leaf development stage, four weeks post-germination, were used for all experiments.

### Insect Material

In late August 2022, a colony of *L. decemlineata* was established in the laboratory by collecting adult beetles from fields at the Oregon State University Hermiston Agricultural Research and Extension Center (45.8404° N, 119.2895° W). Beetles were placed in modified Sterlite (19 L × 16.5 W × 11 H cm) storage containers. Containers were lined with corrugated paper, which was added to absorb excess moisture. In the laboratory, the collected adults were promptly transferred to bug dorms (BugDorm 2F120, Megaview, Taipei, Taiwan). Adults were kept in the laboratory at 27 ± 1 °C, 14:8 L:D, 75% ± 10% RH, and maintained on potato plants (var. “Russet Burbank”); potato plants were replaced weekly. The colony was inspected daily for egg masses. Egg masses were collected and placed onto new potato plants in a separate bug dorm to expand the colony. Fully developed larvae (L4) were allowed to pupate in the potting medium. Three to four days later, pupae were removed from the soil by sifting potting media. Pupae collected were moved to a new bug dorm to restart a colony. F_2_ populations were used in all our experiments. Individual adults of the same age were removed from the colony and placed into individual 50 mL tubes with moistened cotton 24 h before the experiment. This starvation period was done to help ensure the beetles would be responsive to food stimuli.

### Elicitor Application

Potato plants were brought from the greenhouse to the laboratory and sprayed using a calibrated hand sprayer (Chapin 1.4 L Farm and Field hand sprayer, Gemplers, Janesville, WI). The application was made under a fume hood and wearing PPE. Three plants per treatment were sprayed with Actigard 50WG (Syngenta), an SA-type elicitor at 100 mg/L, Blush 2X (Fine America), a JA-type elicitor, at 2 mL/L, and a control was sprayed with water. Following Rodriguez-Saona et al. (2021), who used the same elicitors on cranberries, each plant was sprayed a total of four times, once from every 90° angle, resulting in complete saturation of foliage. Plants were then allowed to dry under the fume hood for 24 h. Gloves were changed between treatments to avoid contamination.

### Y-tube Olfactometer

Adult host selection was assessed using a Y-tube olfactometer. Four treatments were tested in this experiment. The list of treatments can be seen in Table 1. Following Lightle et al. (2010), a glass Y-tube olfactometer (24 mm diameter) with carbon filtration (Analytical Research Systems, Micanopy, FL) was used in this experiment. A bench vacuum, applying 4 L/min of suction, was attached to the base of the center arm. Two L/min of pressure was regulated along each of the side arms using a dual y-tube olfactometer 4-port air delivery system (model OLFM-ADS-4AFM2C Analytical Research Systems, Gainesville, FL). The experiment was lit from above with uniform diffused lighting. Two 2-L glass jars with pressurized seals were used to hold the vapors emitted by entire plants. These were connected to each side arm of the Y-tube with polyethylene tubing.

**Table 1.** The number of individual adults which made a choice in each trial. The total number of beetles counted are the beetles out of *n* = 40 which were capable of making a choice within the allotted 5 min. Those beetles excluded were those which fell onto their backs and were unable to right themselves within the given time

Trial	Choices			Total counted
Blank vs Control	Blank	Control	No choice	25
	2	15	8	
Soil vs Control	Soil	Control	No choice	24
	3	15	6	
Blush vs Control	Blush 2x	Control	No choice	33
	20	3	10	
Actigard 50WG vs Control	Actigard 50WG	Control	No choice	39
	2	9	28	

Adult beetles were placed into the center arm of the Y-tube and given 5 min to make a choice. A choice was considered when the beetle reached a mesh screen placed approximately ¾ of the way up each side arm. The screen was used to prevent beetles from reaching the plant and interacting with gustatory signals, which might bias measures of residency time. This distance was chosen to ensure that the beetle was continuing towards the olfactory cue as far as they were allowed. The trial was recorded as no choice if the beetle did not reach the mesh screen before the time ran out. If the beetle fell and was unable to right itself, the trial was excluded from the final data set. Each pair of choices was offered to 40 individual beetles of mixed sex (*N* = 160) and the Y-tube arms were rotated every 20 beetles. Treatments in the choice test were switched between the left and right arms and consisted of the following pairs: (i) an empty jar vs. an untreated control plant, (ii) potting soil vs. untreated control plant, (iii) plant treated with Blush 2X vs. untreated control plant, and (iv) plant treated with Actigard 50WG vs. an untreated control plant. Choice tests (i) and (ii) were conducted to rule out any attraction toward anything other than the plants in trials (iii-iv). The choice or lack of choice for each beetle was recorded along with the residency time in which they remained on the mesh screen and the time it took for them to make their choice. Each beetle was used once.

### Data Analysis

Data were analyzed using JMP version 16 (SAS Institute Inc., Cary, NC). Preferences for odors in the olfactometer were tested using Pearson’s chi-square analysis of homogeneity. Choices in the olfactometer were tested using Pearson’s Chi-square with three choices: choice 1, choice 2, or no-choice. Choices between the two arm choices without including the no choice were also tested using Pearson’s Chi-square with a Bonferroni correction. The residency time and time till choice for trials were tested using analysis of variance (ANOVA) and pairwise comparisons were made with Tukey HSD after confirming all model assumptions and removing all no choices. Residency time was defined as the continual time in which the insect remained in contact with the mesh screen, which represented a choice.

## Results

### Behavioral Responses to Plant Odors

**Fig. 1.**
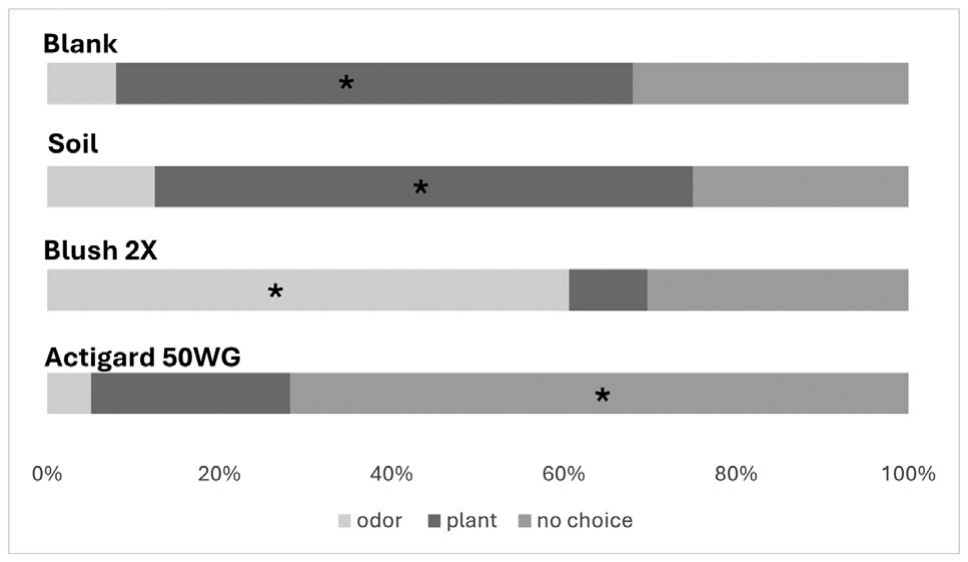
Percentage of beetle’s choices for each trial. Significant majority (*P* < 0.05) compared with control plant is indicated with an asterisk.

During calibration trials, testing a blank chamber or one with potting soil against a control plant, 71% of beetles made a choice within an average of 76 s. When given a choice between the blank chamber or the control plant 88% of beetles chose the control plant. This was comparable to the 83% which chose the control plant over potting soil. The preference for the control plant over the blank or the soil was significant (χ^2^ = 9.9412, *df* = 1, *P* = 0.001616; χ^2^ = 8, *df* = 1, *P* = 0.004678 respectively) (Fig. 1).

When evaluating the attractiveness of plants treated with Blush 2X, a JA-type elicitor, against an untreated control plant, 87% of the beetles chose plants sprayed with Blush 2X over untreated plants. The likelihood of beetles choosing a plant treated with Blush 2X over one treated with just water was significantly different (χ^2^ = 12.565, *df* = 1, *P* = 0.000393; Fig. 1).

However, when offered the choice between a plant treated with Actigard 50WG, an SA-type elicitor, and the plant sprayed with water, 71% of the beetles did not choose while 23% of beetles chose the untreated plant. The likelihood of beetles making no choice when presented with a plant treated with Actigard 50WG or a plant treated with just water was significantly higher (χ^2^ = 28.86, *df* = 2, *P* =< 0.0001; Fig. 1).

### Choice Time

We found no effect of elicitor applications on the time it takes an individual to make a choice between the elicitor-treated plants or an untreated plant (*F* = 2.005, *df* = 4,64, *P* = 0.104; Table 2). However, the residency time with the choice significantly varied (*F* = 9.129, *df* = 4, 64, *P* =< 0.0001; Table 2). Beetles stayed in residence with the control plant longer than all other choices. On average adults were in residence with control plants for 184 s ± 13 while they remained in residence with Actigard-treated plants for only an average of 6 s ± 2. This difference in residence between the control plant and the Actigard-treated plant was significant (*F* = 9.129, *df* = 4.64, *P* = 0.0120, Tukey HSD). There was no significant difference in residency time between control plants and plants treated with Blush 2X.

**Table 2.** Average time (±SE) in seconds for an adult beetle to make a choice and how long they spent in residency with that choice. Significant differences (*P* < 0.05) are indicated between groups a and b

Choice	Time to choice (sec)	Residency
Actigard 50 WG	189 ± 34a	6 ± 2a
Blush 2x	290 ± 97a	105 ± 12a
Blank	76 ± 40a	32 ± 11a
Soil	157 ± 59a	31 ± 5a
Plant	104 ± 11a	184 ± 13b

## Discussion

This study provides evidence that the JA-type elicitor Blush 2X sprayed to “Russet Burbank” potato plants increases its attractiveness to *L. decemlineata* adults. While the SA-type elicitor, Actigard 50WG, may act as a repellent. In this study, we used “Russet Burbank,” which is one of the most intensively cultivated varieties in the USA (USDA NASS 2022). Since the volatile profiles of potato cultivars vary significantly (Agho et al. 2023), our study serves as a baseline test for other potato varieties. When given a choice between two plants, one plant treated with Blush 2X, and the other sprayed with water, of the beetles which made a choice (87%) chose the JA-treated plant and only 10% of beetles chose the control plant. The primary ingredient in Blush 2X is propyl dihydrojasmonate (PDJ), a synthetic derivative of JA. In 1999, Landolt et al. demonstrated that *L. decemlineata* adult females were attracted to plants (var. “Surprise”) sprayed with a gaseous form of another JA derivative, methyl jasmonate. Our data along with Landolt et al. (1999) findings indicate that JA-type elicitors may be generally attractive to *L. decemlineata* adults. This hypothesis is further supported by Gosset et al. (2009), which found a correlation between *L. decemlineata* feeding, the accumulation of JA within potato plants, and an increase in volatiles attractive to *L. decemlineata*. Other studies have also found a general trend of chewing insects, like *L. decemlineata*, being principally affected by JA associated plant immune responses (Liu et al. 2024).

Other studies using foliar applications of the primary ingredient, PDJ, on potato show an increase of shoots’ abscisic acid (ABA) content (Jeong et al. 2021). Abscisic acid is a growth inhibitor that favors storing sugars in tubers and limits vegetative growth (Thornton 2020). Thus, while our data suggests that Blush 2X can be used as an attractant on the cultivar “Russet Burbank,” further studies on the impacts of early-season applications of PDJ on ABA-related metrics like tuberization timing and above-ground biomass are still needed to inform practice.

This study also provided evidence for the SA-type elicitor, Actigard 50WG, being repellent to adult *L. decemlineata*. Close to 71% of beetles made no choice when Actigard 50WG was present, and there was a significant reduction in the residency time of the beetles who chose Actigard. Comparing this to the 60% of beetles that chose the control plant when presented with a blank as the alternative, we see a large decrease in adult beetles locating the host. There are known effector molecules secreted along with *L. decemlineata* saliva which upregulate SA biosynthesis and antagonize the concentration of endogenous JA (Chung et al. 2013). This ecological association with SA may help to explain the repellency as a means to prevent overcrowding. However, several individuals in the SA trials displayed unique behaviors, which confound this potential explanation. Several beetles (35%) in these trials vomited, defecated, or stopped all movement at the crux of the Y-tube. Prior studies on larvae identified these behaviors as antipredator responses (Ramirez et al. 2010). Why volatiles from an SA treated plant would induce such a response is not clear and requires more robust chemical analysis to assess.

Trap cropping is a highly efficacious approach to controlling pests in which the pest is lured to a location for a targeted application of insecticides (Shelton and Badenes-Perez 2006). One key benefit of trap cropping is the ability to reduce the number of insecticide applications needed for control (Hokkanen 1991, Martel et al. 2005). The reduction in selective events on the population is thought to help delay resistance (Martel et al. 2005). Unlike other JA formulations or synthetic male aggregation pheromones, Blush 2X is relatively inexpensive and is currently commercially available. This makes it an ideal option for trap cropping. Additionally, the ability to push *L. decemlineata* away from a crop with Actigard 50 WG while Blush 2X pulls them toward a designated spray area allows for a trap cropping system known as push and pull. Additional research using both these elicitors in a field-based trap cropping system is crucial to integrating the promising findings of this current study into a pest management program.
